# Does Hormone Supplementation With Levothyroxine Improve Hypothyroid Impaired Cognitive Dysfunction?

**DOI:** 10.7759/cureus.17885

**Published:** 2021-09-11

**Authors:** Davuluri Uma, Rizwan Rabbani, Jun Hee Lee, Divya R Gavini, Prutha H Shah, Pousette Hamid

**Affiliations:** 1 Internal Medicine, California Institute of Behavioral Neurosciences & Psychology (CIBNP), Fairfield, USA; 2 Nephrology, California Institute of Behavioral Neurosciences & Psychology (CIBNP), Fairfield, USA; 3 Neurology, California Institute of Behavioral Neurosciences & Psychology (CIBNP), Fairfield, USA

**Keywords:** cognitive impairment, dementia, thyroxine, levothyroxine, hypothyroid

## Abstract

Hypothyroidism is a widespread condition in the United States, affecting approximately 5% of the adult population. Although the clinical use of levothyroxine is well understood, its effect on preventing dementia is not well established. While the exact role of thyroid hormones in the adult brain is unknown, it is apparent that poor thyroid function can lead to mood swings, cognitive impairment, and other psychiatric symptoms. Most studies demonstrate an association between thyroid health and cognition, specifically slow processing of information, decreased effectiveness of executive functions, and lack of learning. This study aims to review the effect of levothyroxine on dementia. We searched electronic databases such as PubMed, Google Scholar, Science Direct, Cochrane, gray literature, and the references of included articles to find relevant articles. Two investigators independently identified eligible studies, screened title/abstract, and extracted data. We identified a total of 319 citations through a database search with six studies (case-control, longitudinal, cross-sectional, randomized controlled trials) meeting the inclusion criteria. Studies with moderate to low risk of bias were evaluated using their respective quality check tools. Five of six studies showed a positive impact of levothyroxine (LT-4) on dementia. According to these studies, the plausible rationale behind the reversal of memory with LT-4 treatment is restoring thyroid-stimulating hormone (TSH), thyroxine (T4) levels, and gamma-aminobutyric acid (GABA) concentrations. People with abnormal thyroid function should be screened for cognitive dysfunction using specific neurocognitive tests and start treatment with LT-4 regardless of symptom presentation. Multi-dose randomized placebo-controlled intervention studies are recommended to assess the effect of LT-4 on lowering the risk of dementia in hypothyroid patients.

## Introduction and background

Hypothyroidism is a common pathologic condition in which the thyroid gland does not produce enough thyroid hormones (thyroxine [T4] and triiodothyronine [T3]) to keep the body's physiologic, metabolic, and cognitive processes functioning accurately [1]. Thyroid hormones concentration in the brain is determined by peripheral T4 levels rather than peripheral T3 [2]. Overt/clinical and subclinical are two classifications of hypothyroidism. Overt hypothyroidism is thyroid hormones below the reference value with elevated thyroid-stimulating hormone (TSH) concentrations. Subclinical hypothyroidism is the biochemical evidence of thyroid hormone deficiency with little to no clinical symptoms [1]. The European Thyroid Association issued guidelines in 2013 that further classified subclinical hypothyroidism into two distinct categories based on TSH cutoffs of 4 to 10 mIU/L or >10 mIU/L [3]. TSH levels rise with age, indicating a propensity for developing subclinical hypothyroidism, as stated in a study conducted in the United States [4,5]. Overt hypothyroidism is becoming more common as people get aged. Treating the patients with subclinical hypothyroidism may prevent the progression to overt hypothyroidism and improve neuropsychiatric signs, cognition, and somatic symptoms [6]. Desiccated thyroid extract was the most widely used thyroid hormone replacement therapy until the 1970s and was replaced by levothyroxine (LT-4) in the latter 20th century [7]. Levothyroxine is a synthetic analog of T4, which gets converted into T3, an active metabolite by deiodinase enzymes.

The estimated prevalence rate of hypothyroidism varies geographically. About 5% of the population in the United States and nine European countries suffer from hypothyroidism [8]. The prevalence of overt hypothyroidism is approximately 0.3% in the U.S. and 0.2-5.3% in Europe [1,9]. The percentage of the population affected varies according to the age group and gender. In the U.S., hypothyroidism affects about 4% of women and 3% of men between 18 and 24, and 21% of women and 16% of men over 74 [10].

Dementia is a progressive deterioration in cognitive functions that makes it difficult to carry out daily activities. Dementia is common when people age, with a prevalence of 10% after 70 years. Greater than 5 million U.S. population is affected with dementia, resulting in a total annual health care cost between $157 and $215 billion [11]. The prevalence of Alzheimer's disease, an age-related dementia, is expected to double by 2050, and that one in 85 people will live with the disease [12,13].

Psychiatric signs are often the first to occur in clinical hypothyroidism [14]. The most common and precocious are neurocognitive alterations (memory and attention difficulties), followed by depression. A lack of attention, abstraction capability, and problem-solving ability can occur during the illness. A lack of diagnosis can lead to severe cognitive deficits and dementia that can become lifelong if not treated [14]. The severity of cognitive impairment remains unknown, although there has been proven evidence of cognitive dysfunction in hypothyroidism.

This study aims to organize a systematic review of all available literature to obtain up-to-date evidence on the clinical efficacy of levothyroxine in lowering the risk of dementia and emphasize the importance of levothyroxine in treating hypothyroidism to avoid or minimize the risk of dementia.

## Review

Methodology

Search Strategy

We referred to Preferred Reporting Items for Systemic Review and Meta-Analyses (PRISMA) guidelines [15] for this systematic review. An electronic search of PubMed, Google Scholar, Science Direct, Cochrane, and gray literature to find published articles related to the keywords. We screened references from selected articles for additional citations and searched databases using terms from three different search concepts (hypothyroid; levothyroxine; dementia). These search terms were combined using the building block approach or the boolean operators "AND" and "OR," which are as follows: Hypothyroid OR Hypothyroidism; Levothyroxine OR Thyroxine OR Levoxyl OR Levo-T; Dementia OR Cognitive decline OR Memory impairment. We used an advanced search strategy and combined all keywords to find articles in the PubMed database.

Eligibility Criteria and Study Selection

Two researchers (UD and RR) independently reviewed the title and abstract of each article to determine eligibility. Following inclusion criteria were used:

(1) Free full texts available

(2) Published in the last 10 years

(3) Studies involving adult human population irrespective of gender or study location

(4) English language

Exclusion criteria were:

(1) Studies involving pregnant women and children

(2) Animal studies

(3) Editorials/journals, the consensus document

(4) Irrelevant articles

For the analysis and data extraction, we finalized the free full-text papers that met the above requirements.

Quality Assessment

For systematic reviews and meta-analyses, we used the Assessment of Multiple Systematic Reviews (AMSTAR) questionnaire, Cochrane risk of bias assessment tools for clinical trials, the New Castle-Ottawa questionnaire for observational studies, and the Scale for the Assessment of Narrative Review Articles (SANRA) for traditional reviews. We did not consider studies that were poor in quality.

Data Extraction

Two authors screened the articles based on the inclusion and exclusion criteria. The authors developed a data extraction spreadsheet and included the following information: author, publication year, journal, study design, sample size, conclusion, cognitive domains, and respective measures.

Results

Overview of Included Studies

**Figure 1:**
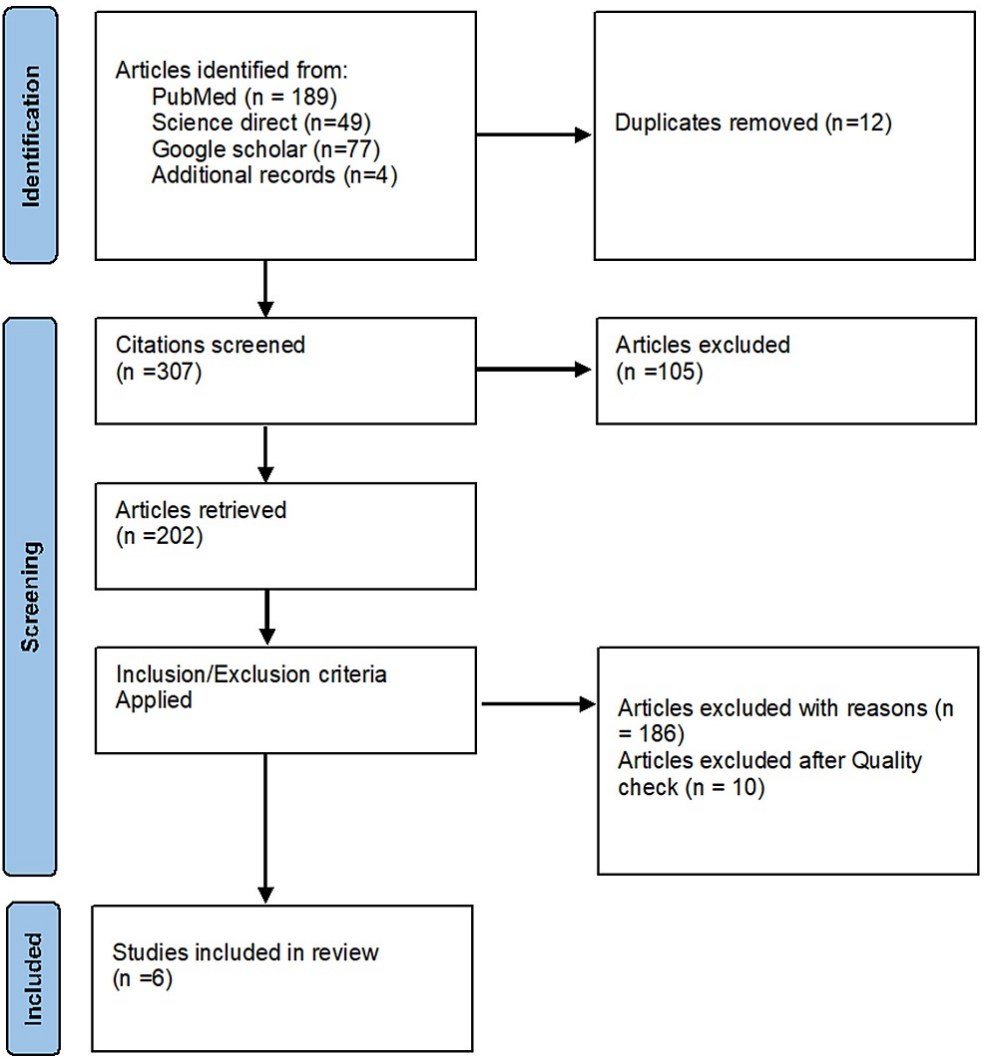
PRISMA flow diagram PRISMA: Preferred Reporting Items for Systematic Reviews and Meta-Analyses [15]

We reviewed a total of 319 articles related to our keywords (search terms). All database references were formatted using Endnote (Clarivate Analytics, Philadelphia, USA), and 12 duplicates were removed using a Microsoft Excel spreadsheet (Microsoft Corporation, Redmond, USA). A total of 307 articles were retrieved for title/abstract screening. After applying inclusion/exclusion criteria, we ended up with 202 articles and excluded 186. We used study-specific quality assessment tools to finalize six articles for data extraction. Figure 1 shows the selection process PRISMA flow chart.

Study Characteristics

Table 1 and Table 2 outline the key features of the six included studies. One case-control study [16], one longitudinal study [17], one cross-sectional study [18], one case report [19], and two randomized clinical trials [20,21] related to the topic. All of these papers were published between 2011 and 2021. Six studies with a total population of 976 people were included.

**Table 1:** Characteristics of the included studies T4- Thyroxine, T3- Triiodothyronine, LT-4- Levothyroxine, TSH- Thyroid Stimulating Hormone, GABA- gamma-aminobutyric acid, RCT- Randomized controlled trials

Author	Year	Journal	Study design	Sample size	Conclusion
Goyal et al. [16]	2018	Indian J Physiol Pharmacol	Case control	30 hypothyroid vs 30 euthyroid controls	Hypothyroidism causes a significant reduction in cognitive skills, and treatment has a substantial effect on cognitive function restoration. In comparison to T3 and T4, this decrease in cognition is better linked with TSH levels.
Liu et al. [17]	2020	Science Direct	Longitudinal study	18	L-T4 treatment reverses the neuropsychiatric impairments caused by abnormal GABA+ and hypothyroidism.
Prashanth et al. [18]	2019	Google Scholar	Cross sectional study	82	Dementia due to hypothyroidism is reversible and it should be treated with thyroid hormone supplementation
Trachtenberg et al. [19]	2012	Brazilian Journal of Psychiatry	Case report	-	Rapid improvement of neuropsychiatric symptoms with levothyroxine replacement.
Aghili et al. [20]	2012	PubMed-Archives of Medical Science	RCT	60	The memory quotient improved significantly after levothyroxine therapy compared to placebo-treated participants.
Stott et al. [21]		PubMed- The New England Journal of Medicine	RCT	737	Replacement of LT-4 has no effect on executive dysfunction.

**Table 2:** Cognitive Domains and Scales used in the included studies MMSE- Mini-Mental Status Examination, DSST- Digital Symbol Substitution Test, LCT- Letter Cancellation Test, TMT- Trail Making Test Parts A&B, MoCA- Montreal Cognitive Assessment, ACE-R- Addenbrooke’s Cognitive Examination Tool, WMS-CR- Weschler Memory Scale-Chinese Revision, μg- Micrograms, NR- Not reported

Source	Cognitive Domain	Measures	Dose of L-Thyroxine	Pre-treatment	Post-treatment
Goyal et al. [16]	Attention, calculation, recall and language, visuomotor coordination, concentration, visual scanning, and rapid response activation and inhibition.	MMSE, DSST, LCT and TMT	NR	Affected	Improvement seen
Liu et al. [17]	Memory	MoCA, WMS-CR	NR	Affected	Rise in scores
Prashanth et al. [18]	Attention, registration, orientation, memory, fluency, language and visuospatial	ACE-R	NR	Affected	Cognitive Mean score Improved
Trachtenberg et al. [19]	Memory	Mini mental scale	100 μg	Affected	Improved
Aghili et al. [20]	Memory	Wechsler Memory Scale	100 μg	Affected	Scores improved
Stott et al. [21]	Execution	Letter digit coding test	50 μg titrated to 25 μg	Affected	No change

Intervention

Two studies investigated levothyroxine replacement with placebo on hypothyroid patients [20,21]. The dose was titrated until the suppression of TSH with the same proportion of dose adjustments in the placebo. Two studies compared pre- and post-treated hypothyroid patients with age and sex-matched euthyroid controls [16,17]. Two studies assessed the post-treatment effect on hypothyroid patients without controls [18,19]. Thyroid function tests were conducted on all patients and controls before and after the replacement of LT-4 in all studies [16-21]. Cognitive functioning was evaluated using Mini-Mental Status Examination (MMSE) [16,19], Addenbrooke’s Cognitive Examination Tool (ACE-R) [18], Digital Symbol Substitution Test (DSST) [16], Letter Cancellation Test (LCT) [16], Trail Making Test Parts A&B (TMT) [16], Montreal Cognitive Assessment (MoCA) [17], Weschler Memory Scale-Chinese Revision (WMS-CR) [17], Weschler Memory Scale (WMS) [20] and Letter Digit Coding Test [21].

Participants

Patients with newly confirmed hypothyroidism were included in three studies [16-18]. Three studies included people patients with a history of hypothyroidism [19-21]. Over half of those participants involved in the studies were women. Most studies included individuals over 18 and under the age of 65, except for two studies where individuals were over 65 [19,21]. Patients who were already on levothyroxine were excluded from the studies.

Duration of Studies

The study duration was between one to 12 months [16-21].

Outcome

Five of the included studies reported an improvement in at least one of the domains of cognitive function [16-20], whereas one study [21] found no statistically significant improvement.

Discussion

This systematic review examined the association between hypothyroidism and cognitive impairment. We also reviewed and analyzed the effect of thyroid hormone replacement on cognitive impairment.

The thyroid hormone has an outstanding role in neuron regulation in the adult brain. Thyroxine (T4) is secreted in higher concentrations compared to triiodothyronine (T3). T4 gets converted to T3 via deiodinase (5′D-II deiodinase) enzyme [22]. Minimal change in the concentration of T4 and T3 within the brain may severely impact behavior. Thyroid hormone has many receptors in the hippocampus and plays a significant role in neurogenesis, cell proliferation, and differentiation of granule cells [23]. A few animal studies reported a 30% reduction in proliferating neurons in the hippocampus without thyroid hormone [24]. LT-4 replacement helps restore these neurons and signaling molecules required for neuronal synaptic plasticity [24,25]. Neuronal synaptic plasticity, also called long-term potentiation, is a crucial mechanism involved in learning and memory [25,26]. Several imaging studies showed objective evidence of decreased hippocampal volume with deteriorated memory in hypothyroid patients [27,28]. 

Hypothyroidism is widely known to be linked to neuropsychiatric symptoms. These symptoms refer to a wide range of emotional and cognitive instability faced by changes in the brain due to various circumstances, including the significant impact of thyroid disease, hormone deficiency in brain tissue, and disturbances in neurotransmission (noradrenergic, GABAergic and serotonin) [29,30]. The clinical presentation includes cognitive, attention, and mood disorders such as depression, which in many cases point to hippocampal dysfunction [24]. Cognitive impairments include poor recall, slow thought process, acalculia, impaired motor learning, and visuospatial abilities leading to pseudo dementia [30-34]. Memory and executive function are the most typically impaired domains [35,36].

Acetylcholine plays a role in the learning and memory process, while gamma-aminobutyric acid (GABA) is involved in mood and cognition regulation [17,37]. Hypothyroidism alters the expression of synaptic proteins involved in acetylcholine release [37]. One of the mechanisms by which LT-4 recovers cognitive function could be increased cholinergic activity [38]. The thyroid and GABA systems are both regulated reciprocally. GABA inhibits the release of TSH from the pituitary gland, while thyroid hormones affect multiple components of the GABA system. Thyroid hormone deficiency may cause disruptions in the GABA synthesizing enzyme, resulting in lower GABA content in the hippocampus [17,39]. An experimental study found a 5% increase in GABA levels after a 6-month treatment with LT-4 and a positive correlation of GABA in the medial prefrontal cortex with memory function [17].

Study Comparison

All review articles included in the study found a definite association between hypothyroidism and cognitive function. Five out of six studies reported positive results on thyroid hormone replacement and reversal of cognitive dysfunction [16-20]. One out of six studies inferred that LT-4 does not affect executive function [21]. After a 12-month trial on an older population, Stott et al. observed no effect on executive function as measured by Letter Digit Coding Test. They concluded that levothyroxine did not show any impact on older people with subclinical hypothyroidism. However, the author also mentioned that a higher thyrotropin target could be why there is no beneficial effect [21]. Goyal et al. observed significant improvement in various domains of cognition, including executive function [16]. Prasanth et al. specified in their study that TSH levels correlate negatively with cognitive function and worsens as TSH levels rise [18]. According to Mulat et al., TSH increases the risk of cognitive impairment by 30% for every unit rise [40]. Few studies also linked decreased TSH levels after treatment to be one reason for restoring cognitive function [16,18,20].

Data from an extensive (n = 5865) cross-sectional community-based investigation evaluating cognitive performance in people aged 65 and up have found no link between subclinical thyroid disease and cognition or depression even though a smaller study of 1047 UK patients aged 64 and up found a link between TSH levels and cognitive performance [41,42]. In a cross-sectional study, using multivariable logistic regression analysis, TSH values (adjusted odds ratio [AOR] = 1.3, 95% CI (1.1, 1.6)) showed a significant association with hypothyroid-associated cognitive impairment [40]. A study comparing TSH levels in an older Korean population (≥ 65 years) reported that low TSH levels (hyperthyroidism) are associated with cognitive impairment rather than high TSH levels (hypothyroidism) [43]. Another study with a two-year follow-up revealed that higher TSH levels are linked with poor performance in MMSE and performed better with TSH normalization [42,44].

Several studies showed positive results similar to our study. Levothyroxine helps in the process of treating hypothyroid-impaired cognitive dysfunction. Aghili et al. reported significant improvement in memory skills in the intervention group compared to the control group in a survey of 60 subclinical hypothyroid patients. The comparison was drawn between the pre- and post-treatment memory scores in both intervention and control groups. The intervention group's memory quotient increased by 9.3 points, while the control group's increased by 3.23 points [20]. A trial of levothyroxine replacement in subclinical hypothyroid individuals reported improvement in verbal fluency, visual and total memory scores [17-19,35,44-47]. These findings are per a study that indicated increased T4 levels result in better verbal performance [12]. Twenty-one hypothyroid and 17 subclinical hypothyroid patients who underwent several neuropsychological tests at baseline, 3 and 6 months were compared with normal subjects. LT-4 replacement improved verbal memory in both overt and subclinical hypothyroid patients; however, only the subclinical group restored spatial memory, while the overt group remained impaired [35]. Imaging studies revealed levothyroxine's reversible effect on cerebral blood flow, brain function, cortical excitability, and brain metabolic activity [17,28,48-50]. A case report on an 81-year-old male patient with memory impairment and behavioral disturbances showed rapid improvement in neuropsychiatric symptoms after levothyroxine replacement. Mini-Mental Score increased from 15 (admission) to 19 (after 10 days) and 21 (40 days after the treatment) with the reversal in memory and orientation [19]. Thyroxine replacement improved spatial learning ability by indicating the LT-4 additive effect on cognitive function, possibly by increasing cholinergic activity [38].

On the other hand, some studies indicate mixed or null findings by levothyroxine replacement on cognitive function. Although thyrotropin levels were restored after a 12-month thyroxine replacement in adults with subclinical hypothyroidism, there was no significant improvement in hypothyroid symptoms. The replacement did not affect executive function (processing speed) as measured by the Letter-Digit Coding Test [21]. Subjects were given LT-4 performed poorly on one of the working memory tests (N-Back) and the motor learning test (Pursuit Rotor) [32]. A double-blind placebo-controlled randomized trial conducted in the United Kingdom found no evidence of significant changes in any of the cognitive domains following LT-4 replacement [51]. Few studies reported partial recovery [33,52-54], and even fewer concluded that replacement either slows or stops the progression rather than restoring [53].

However, hormone replacement could also lead to adverse effects. According to Samuels et al., his cross-sectional study of 20 euthyroid and 34 treated hypothyroid patients with LT-4 showed decreased health status, psychological function, working memory, and motor learning compared to euthyroid controls [32]. According to an online survey of hypothyroid patients, 60% of those who received an LT-4 drug experienced increased memory problems [55].

Limitations

Our study has multiple limitations, one of which is the exclusion of non-English publications. In addition, each study used different doses of levothyroxine with specific tools for evaluating cognition. The majority of published articles assessed only limited aspects of cognitive function. The risk of dementia is higher for those who are above the age of 65 years. Most of the study population was over 60 years of age.

## Conclusions

The findings of this review imply that levothyroxine helps in the reversal of dementia/cognitive impairment associated with thyroid dysfunction, which in turn reduces the severity of neuropsychiatric symptoms, hospitalizations, and associated costs. However, differences between study results on the effect of levothyroxine on cognitive dysfunction may be due to different tools used to assess cognitive function, the dose of levothyroxine, age, and sample size of the population in the study.

Recommendations

Additional multi-dose randomized placebo-controlled intervention studies are needed to assess the effect of levothyroxine on lowering the risk of dementia in hypothyroid patients. People with abnormal thyroid function should be screened for cognitive dysfunction using specific neurocognitive tests and start treatment with levothyroxine regardless of symptom presentation.
